# A Medical Service for All

**Published:** 1920-12-25

**Authors:** N. Howard Mummery

**Affiliations:** The Federation of Medical and Allied Societies, 5 Vere Street, W. 1


### THE EDITOR'S LETTER-BOX.

**Correspondence n all subjects is invited, but we cannot in any way be responsible for the opinions expressed by our correspondents, who must give their name and address as a guarantee of good faith, but not necessarily for publication. Correspondents are reminded that brevity of style and conciseness of statement greatly facilitate early insertion.**

### "A Medical Service for All."

*To the Editor of* The Hospital.

Sir,—Will you allow me to draw attention to the present *impasse* in the organisation of medicine? The situation is unworthy of any body of men, and least of all is it worthy of our profession.

Excluding for the moment the Royal Colleges, there are three organisations in the medical profession interested in what may be called, for want of a better term, medical politics, namely :—

The British Medical Association, an organisation of individual medical men with a membership of some 20,000 (including those resident in the Colonies and abroad), or, roughly, 52 per cent. of registered practitioners.

The Federation of Medical and Allied Societies, an organisation of societies at present uniting rather more than 18,000 medical practitioners in Great Britain and some 90,000 of those in the allied dental, pharmaceutical, nursing, and midwifery professions.

The Medico-Political Union, an organisation of individual medical practitioners on trade-union lines, with a membership of some 3,200.

The first and third of these exist to further the interests of doctors alone. The Federation exists to advance the interests of the general public by providing the means of sounding the collective opinion of the various sections in the medical profession, in the allied professions, and in the community at large. As such it neither aspires to leadership nor to control of its constituent bodies.

The View of the Minister of Health.

Dr. Addison's position is difficult, but conclusions may be drawn from his recent statements.

On November 1, 1920, he told a deputation of the B.M.A. that "he was not going to consult half-a-dozen different bodies each claiming to represent the profession." He also said, "If you want us to consult you, get busy, and set up some machinery whereby you can readily be consulted." He pointed out that "in each area there might be a branch of the B.M.A., but there were also other associations and societies as well."

On December 9, 1920, Dr. Addison received a deputation from the Medico-Political Union, and took the opportunity to make the following remarks : "Since the early days of the Ministry of Health there have been a large number of deputations of medical men on important subjects which the Department has under consideration. In such circumstances the voice of a united medical profession, speaking not of its own interests primarily, is most urgently required, but has not yet been obtained. There is no lack of those who say they represent the profession as a whole, but there is no one body which even claims to represent all interested organisations, though some aspire to. If you wish to procure attention for your views you must agree together first. In no other way is it possible for your views to be completely represented in the councils of those concerned with the reconstruction of our public health system."

The View of the British Medical Association.

To quote the words of the Medical Secretary and other stalwarts of the Association, who seem to constitute an oligarchy in an otherwise democratic body : "The Association is recognised as the mouthpiece of the profession." "There is only one organisation in the medical profession—namely, the Association." "The Association could, if loyally supported by the whole profession, do all that the Federation aims at doing."

On December 15, 1920, the Central Council of the Association rejected the recommendation of its own Ministry of Health Sub-Committee, which body advised co-operation with the Federation.

The View of the Federation.

Desiring only to meet the needs of the community and the allied professions, the Federation has adopted a policy of neutrality and conciliation. It claims that by no other means than those it provides can the collective voice of a great profession and its allies in the public service be made available in the councils of the nation. Amongst the forty-five organisations co-operating at the present time may be mentioned the Royal Society of Medicine, the British Science Guild, the Medical Women's Federation, Medico-Psychological Association, Association of Medical Officers of Health, British Dental Association, Pharmaceutical Society, College of Nursing, Royal British Nurses' Association, and Midwives' Institute, to mention a few of the larger bodies only. And as all the allied professions are fully represented, it requires only the co-operation of the British Medical Association and the Medico-Political Union to make adequate representation of medicine possible. Is not this a goal worth some personal sacrifice?—I am, Sir, yours truly,

N. Howard Mummery,

The Federation of Medical and Allied Societies,
5 Vere Street, W. 1.

December 21, 1920.

